# Selective Inactivation of Intracellular BiP/GRP78 Attenuates Endothelial Inflammation and Permeability in Acute Lung Injury

**DOI:** 10.1038/s41598-018-38312-w

**Published:** 2019-02-14

**Authors:** Antony Leonard, Valerie Grose, Adrienne W. Paton, James C. Paton, David I. Yule, Arshad Rahman, Fabeha Fazal

**Affiliations:** 1 Department of Pediatrics, Lung Biology and Disease Program, University of Rochester School of Medicine and Dentistry, Rochester, New York 14642 USA; 2 Research Centre for Infectious Diseases, Department of Molecular and Biomedical Science, University of Adelaide, Adelaide, South Australia Australia; 3 Department of Pharmacology & Physiology, University of Rochester School of Medicine and Dentistry, Rochester, New York 14642 USA

## Abstract

The role of Endoplasmic Reticulum Chaperone and Signaling Regulator BiP/GRP78 in acute inflammatory injury, particularly in the context of lung endothelium, is poorly defined. In his study, we monitored the effect of SubAB, a holoenzyme that cleaves and specifically inactivates BiP/GRP78 and its inactive mutant SubA_A272_B on lung inflammatory injury in an aerosolized LPS inhalation mouse model of acute lung injury (ALI). Analysis of lung homogenates and bronchoalveolar lavage (BAL) fluid showed that LPS-induced lung inflammation and injury were significantly inhibited in SubAB- but not in SubA_A272_B-treated mice. SubAB-treated mice were also protected from LPS-induced decrease in lung compliance. Gene transfer of dominant negative mutant of BiP in the lung endothelium protected against LPS-induced lung inflammatory responses. Consistent with this, stimulation of endothelial cells (EC) with thrombin caused an increase in BiP/GRP78 levels and inhibition of ER stress with 4-phenylbutyric acid (4-PBA) prevented this response as well as increase in VCAM-1, ICAM-1, IL-6, and IL-8 levels. Importantly, thrombin-induced Ca^2+^ signaling and EC permeability were also prevented upon BiP/GRP78 inactivation. The above EC responses are mediated by intracellular BiP/GRP78 and not by cell surface BiP/GRP78. Together, these data identify intracellular BiP/GRP78 as a novel regulator of endothelial dysfunction associated with ALI.

## Introduction

Acute lung injury (ALI) is a common cause of respiratory failure in critically ill patients with a mortality rate of 38.5%^1^. ALI can be precipitated by either direct insults such as pneumonia, aspiration or via indirect insults such as sepsis and multiple trauma, to the lungs^2^. The vascular endothelium forming the innermost lining of all pulmonary blood vessels is the major barrier that protects air spaces against vascular fluid entry. Upon microbial infection, products such as lipopolysaccharides (LPS) from Gram-negative bacteria are released into the pulmonary circulation where they interact with lung vascular endothelial cells (EC) lining the blood capillaries. Vascular EC exposed to bacterial toxins secrete inflammatory and chemotactic substances, express adhesion molecules and demonstrate loss of barrier integrity^1^. Disruption of pulmonary endothelial barrier function and acquisition of a proinflammatory phenotype are among the major pathogenic features of ALI^3,4^.

Activation of the transcription factor NF-κB is a key mechanism responsible for the acquisition of the proinflammatory phenotype in the lung. Activated NF-κB converts the otherwise “antiadhesive” lung vascular endothelium into a “proadhesive” one via activation of adhesion molecules (ICAM-1, VCAM-1), cytokines (TNF-, IL1β, IL-6), and chemokines (IL-8 and MCP-1), which in turn facilitates the adhesion and subsequent transendothelial migration of inflammatory cells, particularly neutrophils (polymorphonuclear leukocytes [PMN]) into the alveolar air space^5–10^. The mechanism underlying increased lung endothelial permeability involves disruption of VE-cadherin homodimers, the key components of adherens junction (AJs). In addition to VE-cadherin disassembly, actin-myosin interaction is critical to EC barrier disruption caused by proinflammatory agonists^11–15^. Together, these events (NF-κB activation and VE-cadherin disassembly) contribute to ALI pathogenesis^16–20^.

The endoplasmic reticulum (ER) is a major site for the synthesis and maturation of secretory and membrane proteins and therefore plays essential roles in physiological regulation of many cellular processes^21^. BiP/GRP78 (Binding Immunoglobulin Protein/78-kDa glucose-regulated protein), also referred to as heat-shock protein A5 (HSPA5), is primarily regarded as an ER chaperone involved in protein folding and assembly, Ca^2+^ homeostasis, and regulating ER stress signaling. Disturbances in ER homeostasis, due to glucose deprivation, disturbances in Ca^2+^ homeostasis, viral and bacterial infections, can cause imbalance in the luminal flux of the newly synthesized unfolded or misfolded peptides resulting in a condition known as ER stress^22^. To combat ER stress an adaptive mechanism called the unfolded protein response (UPR) is activated. One of the pathways activated under UPR involves expression of ER chaperone BiP/GRP78 to assist in proper protein folding, maintain chaperone homeostasis, and support cell survival. However, recent studies have shown that BiP/GRP78 not only resides in the ER lumen, but also outside the ER (cytoplasm, mitochondria, nucleus, and plasma membrane), and performs different functions in different cellular compartments^23^. Intracellular BiP/GRP78 regulates ER stress-induced signaling and apoptosis, whereas cell surface BiP/GRP78 acts as receptor for both viral entry and for proliferation and apoptotic signaling. Studies have shown that BiP/GRP78 is also critical to embryonic development, aging, insulin-mediated signaling and pathological conditions, including cancer, diabetes, obesity and neurological disorders^24–27^. However, the role of BiP/GRP78 in *acute* inflammatory injury, particularly in the context of lung endothelium, remains largely unknown. In order to ascertain the role of BiP both in primary endothelial cells and in a LPS inhalation murine model of ALI, we used Subtilase cytotoxin (SubAB), the prototype of a family of AB5 cytotoxins produced by Shiga toxigenic *Escherichia coli*, or its isolated catalytic A subunit (SubA), that specifically cleaves BiP/GRP78 between a dileucine motif (Leucine_416/417_), resulting in inactivation of BiP^28,29^. Our study identifies intracellular BiP/GRP78 as an important mediator of lung vascular inflammation and injury through its ability, at least in part, to activate EC inflammation and barrier disruption.

## Results

### BiP/GRP78 is a critical mediator of lung inflammation and injury

**Figure 1:**
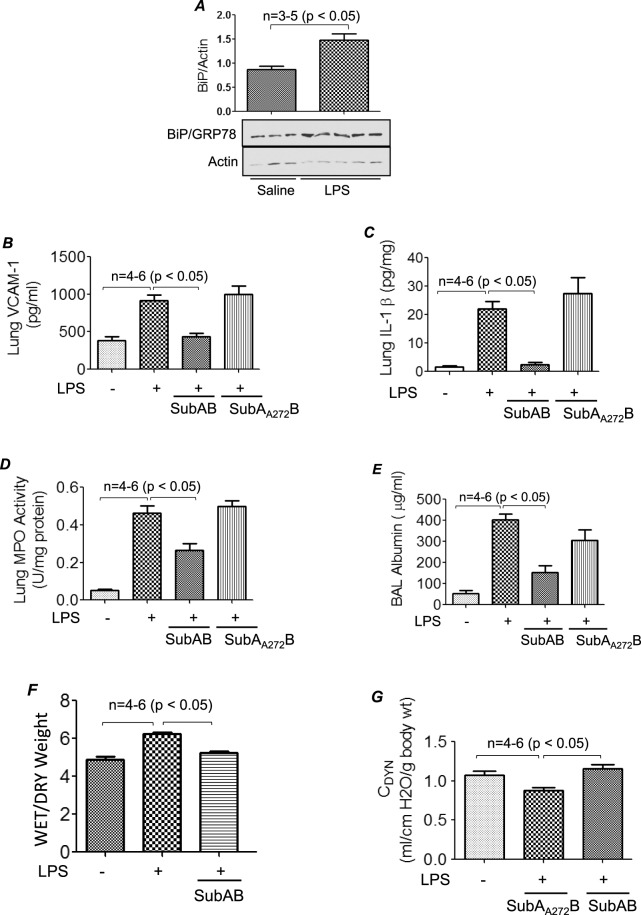
SubAB protects against LPS-induced lung inflammation and injury. Wild type C57BL/6 mice were injected i.p. with SubAB or mutant SubA_A272_B (0.04 mg/kg bodyweight) for 6 h. Mice were then aerosolized with 6 ml of saline alone or saline containing *E*.*Coli* LPS (0.5 mg/ml) for 30 min. Eighteen hours after LPS challenge, lung homogenates were analyzed for levels of BiP/GRP78 (**A**), proinflammatory mediators VCAM-1 (**B**) and IL-1β (**C**) by ELISA and neutrophil sequestration by measuring tissue MPO activity (**D**). Bronchoalveolar lavage (BAL) fluids were analyzed for albumin levels (**E**) Lungs were analyzed for wet-to-dry weight ratio (**F**). Live ventilated mice were evaluated for dynamic lung compliance (**G**) using whole-body plethysmograph as described in Materials and Methods section.

In order to test the possibility whether BiP/GRP78 is a critical player in mediating lung vascular inflammation and injury we first determined if BiP expression was regulated in an aerosolized bacterial LPS inhalation mouse model of ALI. A marked increase in BiP/GRP78 expression was observed in lung homogenates of mice treated with LPS (Fig. 1A). No mortality was observed in these mice as they were exposed to a sublethal dose of LPS (0.5 mg/ml; 6 ml). Furthermore mice were treated intraperitoneally (i.p.) with BiP/GRP78 inhibitor SubAB or its inactive mutant SubA_A272_B for 6 h and then challenged with LPS for 16 h. Analysis of lung homogenates from these mice showed that LPS inhalation induced the levels of proinflammatory mediators VCAM-1 and IL-1β; but these responses were strongly inhibited in SubAB treated mice (Fig. 1B,C). In contrast, mice pretreated with SubA_A272_B showed no protection against LPS-induced increase in VCAM-1 and IL-1β levels. Consistent with this, infiltration of neutrophils in the lung, as measured by myeloperoxidase (MPO) activity, was also inhibited in mice treated with SubAB but not in the mice treated with SubA_A272_B (Fig. 1D). Analysis of the bronchoalveolar lavage (BAL) showed that LPS substantially induced albumin levels, an indicator of microvascular leakage; this response was inhibited by SubAB but not by SubA_A272_B (Fig. 1E). We next determined the effect of SubAB and SubA_A272_B on lung wet-to-dry weight ratio, an important indicator of lung tissue edema, and found that LPS-induced lung tissue edema was protected in the presence of SubAB (Fig. 1F). These data led us to determine if SubAB is also effective in improving lung function in mice challenged with LPS. To this end, we assessed the dynamic lung compliance in live ventilated mice and found that LPS-induced decrease in lung compliance was protected in mice pretreated with SubAB but not in mice pretreated with SubA_A272_B (Fig. 1G). These data establish that BiP/GRP78 as a critical determinant of lung inflammatory injury in mice.

### Endothelial BiP/GRP78 contributes to lung vascular inflammation

**Figure 2:**
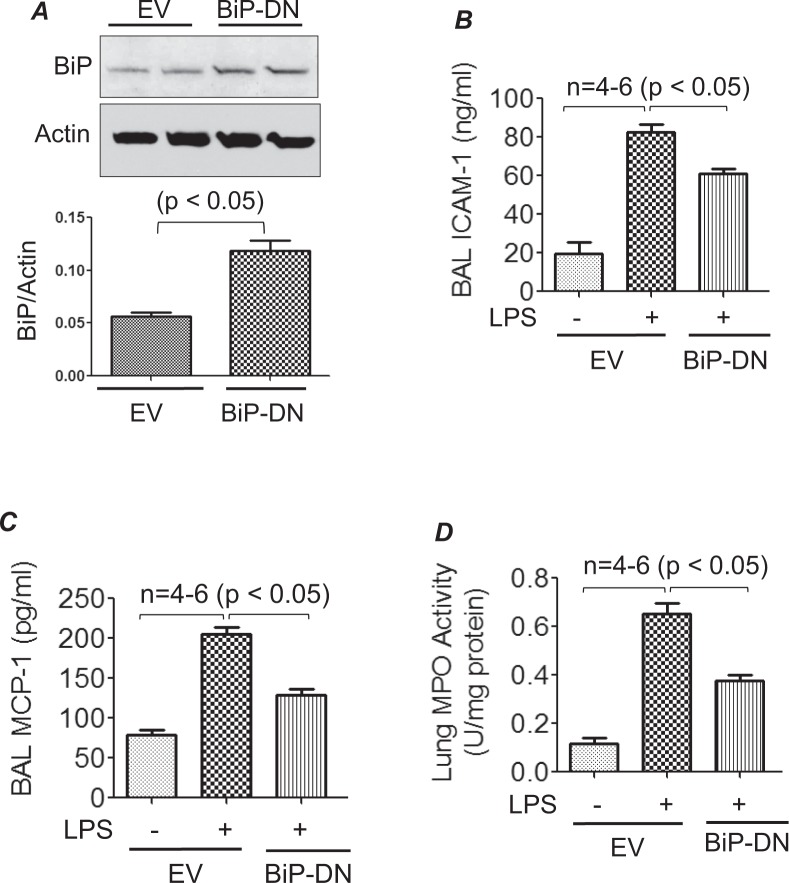
Cationic liposome mediated gene transfer of BiP/GRP78-DN dampens LPS-induced lung injury. WT C57BL/6 mice were injected i.v. with empty vector (EV)/liposome or dominant negative mutant BiP/GRP78 (BiP/GRP78-DN)/liposome complex. After 72 h, mice were aerosolized with 6 ml of saline alone or saline containing *E*.*Coli* LPS (0.5 mg/ml) for 30 min. Sixteen hours later, lung homogenates were analyzed for expression of BiP/GRP78-DN (**A**). BAL fluids were analyzed for ICAM-1 (**B**) and MCP-1 (**C**) levels. Lung homogenates were analyzed for MPO activity (**D**).

In order to understand the role of endothelial BiP/GRP78 in LPS induced lung vascular inflammation, wild type C57BL/6 L mice were transduced with a plasmid expressing a dominant negative mutant of BiP/GRP78 (p-BiP/GRP78-DN) or empty vector (EV) via intravenous injection of cationic liposomes, which primarily targets the lung endothelium for gene transfer^30–32^. Seventy-two hours after the injection, mice were challenged with LPS for 16 h. Lung homogenates were analyzed for expression of the transduced plasmid (p-BiP/GRP78-DN). Increased expression of BiP/GRP78 was observed in mice transduced with p-BiP/GRP78-DN compared to mice transduced with EV (Fig. 2A). Furthermore, BAL fluid was analyzed for the levels of proinflammatory mediators such as ICAM-1 and MCP-1 and for PMN recruitment. Data showed that LPS-induced ICAM-1 and MCP-1 levels as well as PMN infiltration were significantly inhibited in mice expressing BIP/GRP78-DN compared to mice transduced with EV (Fig. 2B–D). Together, these results indicate that endothelial BiP/GRP78 plays a vital role in causing lung vascular inflammation.

### ER stress is critical to EC inflammation

**Figure 3:**
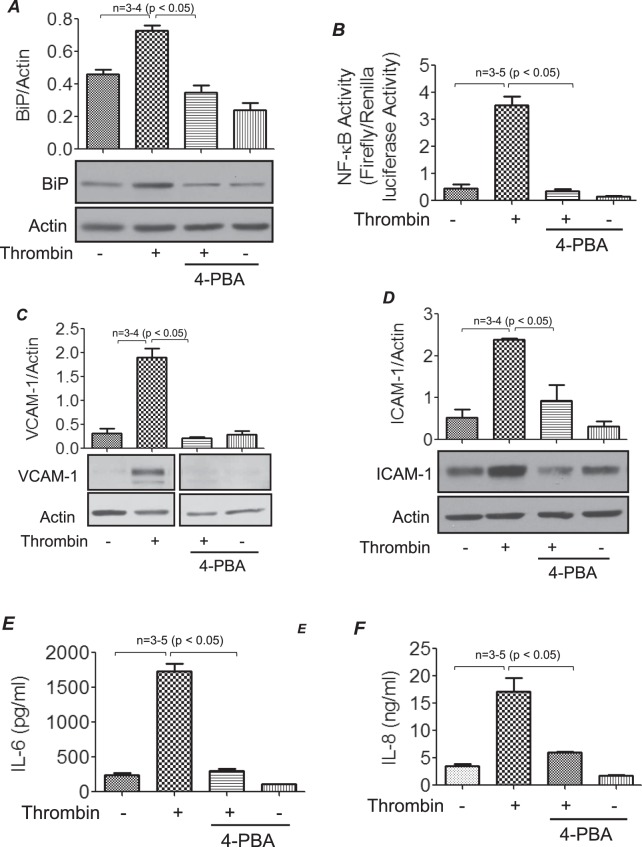
Thrombin-induced inflammatory responses are mitigated in the presence of ER stress inhibitor 4-PBA. HPAEC were pretreated with 4-PBA (10 mM) for 1 h followed by treatment with 5 U/ml thrombin for 6 h. Total cell lysates were probed for BiP/GRP78 (**A**). HPAEC were transfected with NF-κB-LUC and pTKRLUC constructs by DEAE-dextran as described in Methods. Cells were then treated with 4-PBA (1 h) followed by thrombin treatment (5U/ml) for 6 h and cell extracts were prepared and assayed for Firefly and Renilla luciferase activities (**B**). Total cell lysates treated with 4-PBA followed by thrombin treatment were also probed for VCAM-1(**C**) and ICAM-1(**D**) by western blotting and cell supernatants were analyzed for IL-6 (**E**) and IL-8 (**F**) levels by ELISA. The blot in 3 C is cropped from two different parts of the same gel, as shown by white space.

Because BiP/GRP78 is a known mediator of ER stress, we investigated if the ER stress-BiP/GRP78 axis is critical to EC inflammation associated with ALI. We determined if thrombin promotes BiP/GRP78 expression in an ER stress-dependent manner in EC. Stimulation of human pulmonary artery endothelial cells (HPAEC) with thrombin caused an increase in BiP/GRP78 levels and pretreatment of cells with the ER stress inhibitor 4-phenylbutyric acid (4-PBA)^33^ prevented thrombin-induced increase in BiP/GRP78 level (Fig. 3A). We next determined whether the ER stress-BiP/GRP78 axis also mediates thrombin-induced EC inflammation. Results showed that pretreatment of cells with 4-PBA prevented thrombin-induced transcriptional activity of NF-κB (Fig. 3B). Furthermore, a marked reduction in thrombin-induced ICAM-1, VCAM-1, IL-6 and IL-8 levels was noted in the presence of 4-PBA, consistent with its effect on NF-κB activity (Fig. 3C–F). It should be noted that siRNA-mediated knockdown of BiP/GRP78 produced a similar effect on thrombin-induced EC inflammation^22^. Together, these data support a role for ER stress-BiP/GRP78 axis in EC inflammation.

### Intracellular pool of BiP/GRP78 is critical for EC inflammation

**Figure 4:**
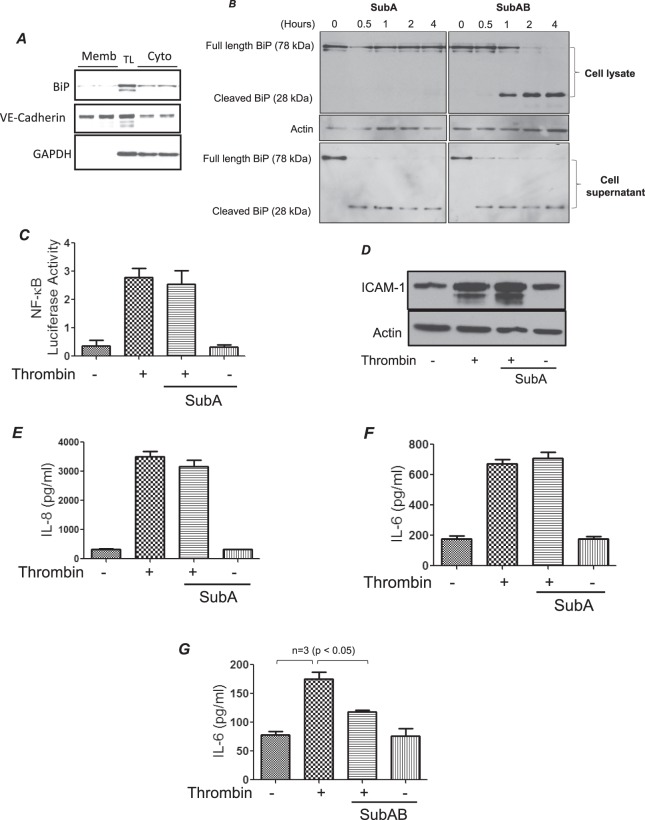
SubA fails to mediate thrombin-induced inflammatory response. Confluent HPAEC were biotinylated using Sulfo-NHS-SS-Biotin, subsequently lysed and labeled cell surface proteins were isolated using streptavidin beads and then separated on an SDS-PAGE and probed for BiP, VE-cadherin and GAPDH (**A**). TL is total lysate. HPAEC were treated with 0.1 µg/ml of SubA and SubAB for the indicated time points. Cell lysates and corresponding cell supernatants were immunoblotted for BiP/GRP78 to monitor the cleavage of BiP/GRP78 by SubA and SubAB (**B**). The blot in 4B is cropped from two different parts of the same gel, as shown by white space. HPAEC were transfected with NF-κBLUC and pTKRLUC constructs by DEAE-dextran as described in Materials and Methods section. Following transfection cells were treated with 0.1 µg/ml of SubA for 3 h and then by 5 U/ml of thrombin for 6 h. Total cell lysates were prepared and assayed for Firefly and Renilla luciferase activities (**C**). Confluent HPAEC were pretreated with SubA for 3 h followed by thrombin treatment (5 U/ml) for 6 h. Cell lysates were probed for ICAM-1(**D**) and cell supernatants were analyzed for IL-8 (**E**) and IL-6 (**F**) by ELISA. HPAEC were pretreated with SubAB for 3 h followed by thrombin treatment (5U/ml) for 6 h. Total cell lysates were probed for IL-6 (**G**) by ELISA.

BiP/GRP78 is shown to be present not only in the ER but in extra-ER regions as well, such as the cytosol and plasma membrane^23^. In order to ascertain the role of intracellular versus cell surface BiP/GRP78 we used two specific inhibitors; SubAB and SubA. SubAB is a serine protease composed of a 35-kDa catalytic A subunit (SubA), and five 13-kDa B subunits. The A subunit contains the catalytic triad Asp_270_, His_271_ and Ser_272_, which is responsible for cleaving BiP/GRP78 between Leucine_416–417_ and thereby inhibiting its function. Mutation of Ser_272_ to Ala_272_ results in a catalytically inactive enzyme (SubA_A272_B). The B subunit mediates binding to glycan receptors on the cell surface and is necessary for internalization and subsequent trafficking of the holoenzyme to the ER. Consequently, SubAB cleaves and inactivates the global pool of BiP/GRP78 (intracellular and cell surface), while SubA only cleaves and inactivates the cell surface BiP/GRP78^28,29^. Importantly, both SubAB and SubA display extreme substrate specificity towards BiP/GRP78^28,29^. Since not every cell type expresses BiP/GRP78 on the cell surface we first wanted to confirm the expression of cell surface BiP/GRP78 in HPAEC. Our data showed that BiP/GRP78 is expressed on endothelial cell surface in low levels and the majority of it resides intracellularly. Next, we confirmed the mode of action of SubAB and SubA towards BiP/GRP78 by western blot analysis (Fig. 4B). As expected, time course analysis showed that pretreatment of EC with SubA resulted in the appearance of cleaved BiP/GRP78 band (~28 KDa) only in cell culture supernatants and not in cell lysates, whereas pretreatment with SubAB generated the 28 KDa cleaved BiP/GRP78 band both in cell culture lysates and supernatants (Fig. 4B). Next, the effect of SubA was analyzed on thrombin-induced EC inflammation. Interestingly, SubA failed to inhibit thrombin-induced activation of NF-κB and expression of ICAM-1, IL-8 and IL-6 (Fig. 4C–F). This is in contrast to the effect of SubAB which inhibits thrombin-induced EC inflammation (Fig. 4G)^22^. These data identify intracellular BiP/GRP78 as an important regulator of EC inflammation induced by thrombin.

### BiP/GRP78 mediate EC adhesivity towards HL-60

**Figure 5:**
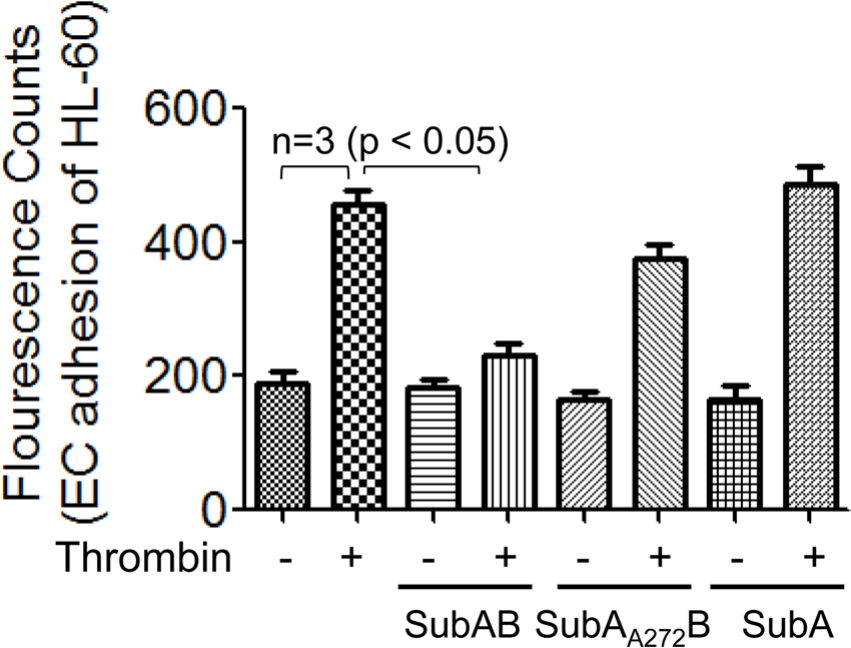
SubAB selectively inhibits adhesion of HL-60 to thrombin activated EC. HPAEC were grown to confluence in 96-well black polystyrene plates. Confluent monolayers were treated with 0.1 µg/ml of SubAB, SubA_A272_B or SubA followed by treatment with thrombin (5 U/ml) for 5 h. HL-60 cells were labelled with 2.5 µM Calcein-AM for 30 min. Calcein-AM labeled HL-60 cells were added to each well for 1 h at 37 °C followed by gentle wash with Phenol Red free DMEM media 4 times. Fluorescence was measured using a fluorescent plate reader at an excitation of 495 nm.

In order to understand the functional relevance of BiP/GRP78 in EC inflammation, we assessed the adhesion of the neutrophilic cell line HL-60 to the activated EC (expressing adhesion molecules upon thrombin treatment)^34^. Results showed that thrombin increased EC adhesivity toward HL-60 cells and that SubAB prevented this response. Unlike SubAB, SubA_A272_B showed no significant effect on thrombin-induced EC adhesivity toward HL-60 cells (Fig. 5). In contrast, SubA failed to inhibit adhesion of HL60 cells to EC stimulated with thrombin (Fig. 5). These data are consistent with the effects of SubAB, SubA_A272_B^22^ and SubA on thrombin-induced expression of proinflammatory genes (Fig. 4D–F) and support a role of intracellular BiP/GRP78 in regulating thrombin-induced EC adhesivity.

### BiP/GRP78 is critical to thrombin-induced EC permeability via VE-cadherin disassembly

**Figure 6:**
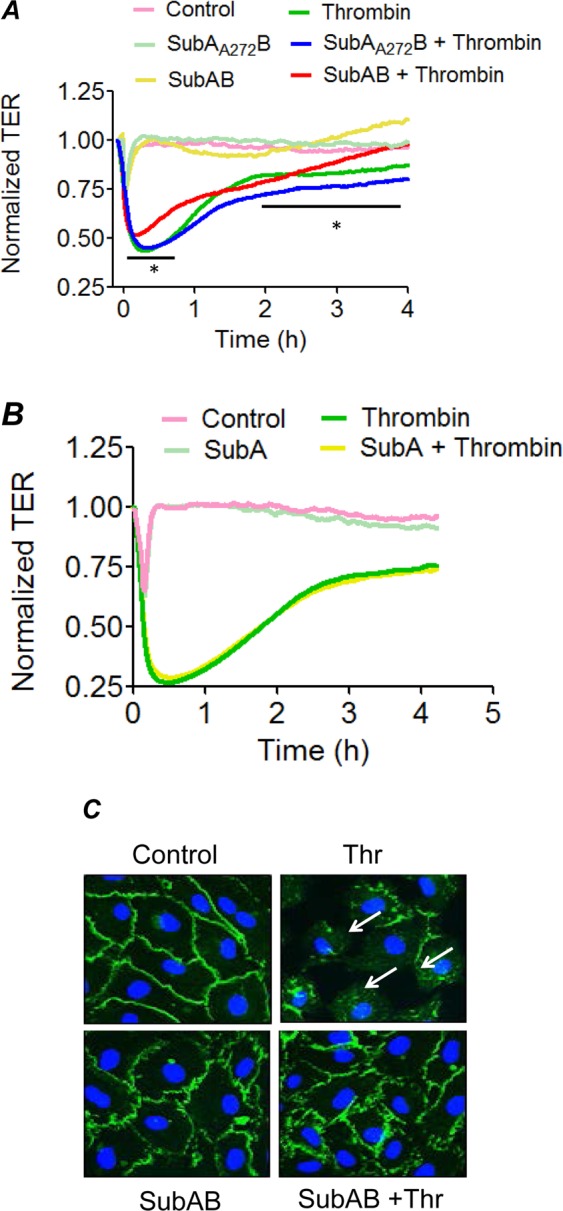
SubAB protects against thrombin-induced endothelial barrier disruption and VE-Cadherin disassembly. Confluent HPAEC grown on gold electrode plates were treated with 0.1 µg/ml of SubAB or SubA_A272_B (**A**) or SubA (**B**) for 3 h and then challenged with thrombin (2.5 U/ml). Real time changes in transendothelial electrical resistance (TER) were measured to monitor endothelial barrier function after thrombin treatment (**A**,**B**). Data are mean ± SE (n = 3–5 for each condition). *Difference between thrombin treated versus thrombin + SubAB treated (p < 0.05). HPAEC grown to confluence on 2% gelatin coated coverslips were treated with 0.1 µg/ml of SubAB for 3 h followed by treatment with thrombin (5 U/ml) for 15 min. Immunofluorescence was performed to visualize VE-cadherin (green) and nuclei (blue) (**C**). Arrows indicate disruption of VE-cadherin staining. Images are representative of 3 experiments.

Because loss of endothelial barrier integrity is another major pathogenic feature of ALI, we next addressed the role of BiP/GRP78 in thrombin-induced EC permeability. Trans-endothelial resistance (TER), which is a real time measurement of EC permeability, was performed after thrombin challenge^35^. Maximal decrease in TER was observed around 0.5 h and the cells gradually recovered to baseline by 2–4 h. Inactivation of BiP/GRP78 by SubAB not only protected against thrombin-induced decrease in TER at earlier time points (0.25–1 h) but also enhanced the recovery after 2.5 h (Fig. 6A). In contrast, SubA_A272_B not only failed to protect against thrombin-induced decrease in TER but also slowed the recovery after 1 h of thrombin challenge (Fig. 6A). These data are consistent with the protective effect of BiP/GRP78 knockdown on thrombin-induced inter-endothelial gap formation^22^. Interestingly, however, unlike SubAB, SubA had no effect on thrombin-induced decrease in TER (Fig. 6B). Together these data indicate a role of intracellular BiP/GRP78 in mediating EC permeability. Next, we determined whether the protection by SubAB against thrombin-induced decrease in TER is mediated via loss of VE-cadherin at AJs. Confocal microscopy revealed that thrombin-induced a decrease in immunostaining of VE-cadherin at AJs, whereas pretreatment with SubAB prevented the response (Fig. 6C). These data identify intracellular BiP/GRP78 as a critical mediator of EC barrier disruption through its ability to induce VE-cadherin disassembly.

### BiP/GRP78 is critical to thrombin-induced Ca2^+^ signaling in EC

**Figure 7:**
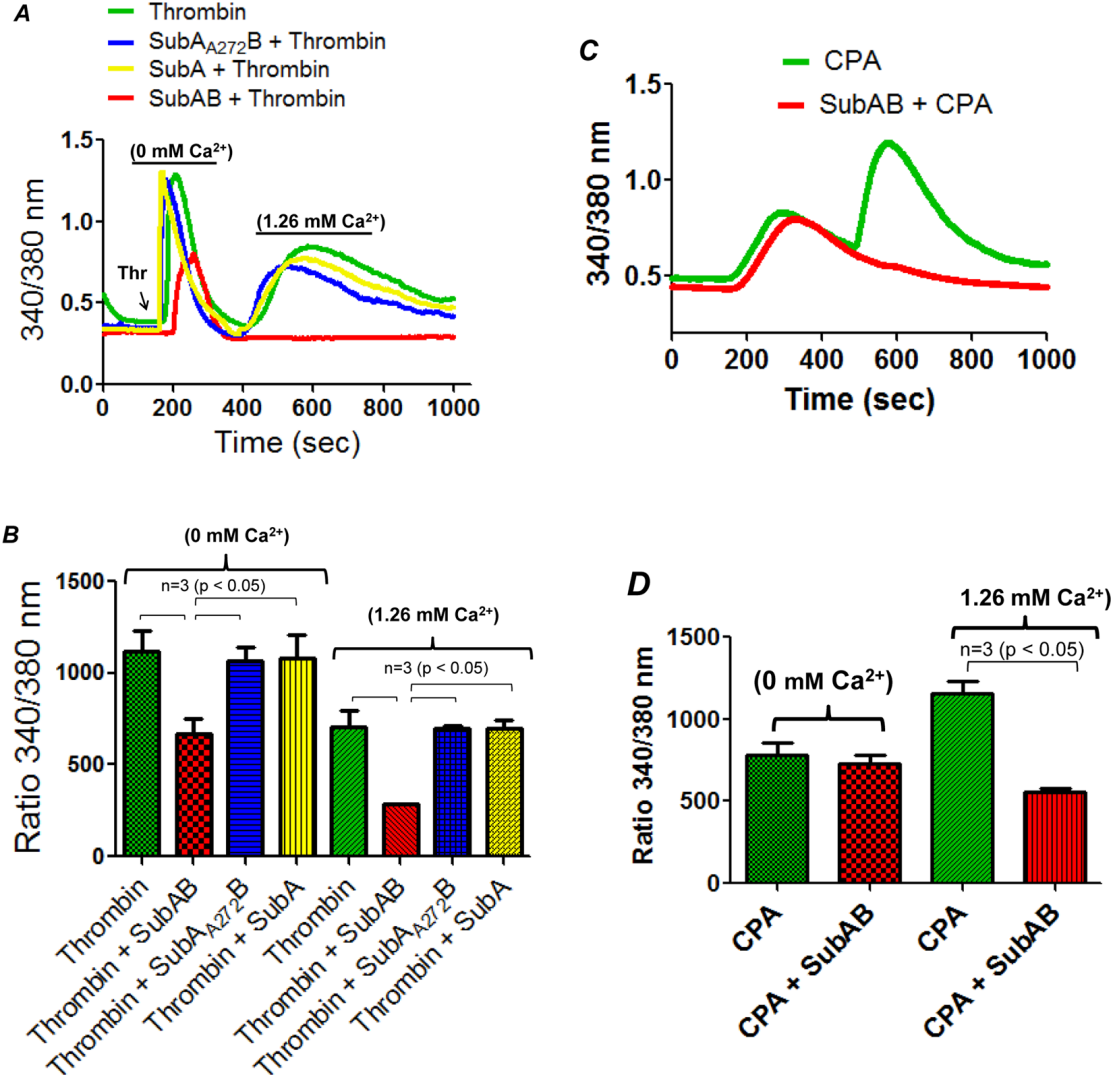
SubAB inhibits thrombin-induced Ca^2+^ release and entry in EC. HPAEC grown to confluence on 25 mm coverslips were treated with 0.1 µg/ml of SubAB or SubA or its inactive mutant SubA_A272_B for 3 h. Cells were then loaded with Fura2-AM for 30 min and washed twice with Ca^2+^ free HBSS buffer and mounted on an inverted microscope. Calcium release from the intracellular stores was determined by perfusing Ca^2+^ free imaging buffer and stimulating cells with thrombin (2.5 U/ml). Store-operated Ca^2+^ entry was measured following addition of 1.26 mM Ca^2+^ (**A**,**B**). HPAEC treated with or without SubAB were loaded with Fura2-AM and stimulated with 30 µM CPA in the absence of extracellular Ca^2+^, to deplete ER Ca^2+^. This was followed by re-addition of 1.26 mM Ca^2+^ to determine Ca^2+^ entry (**C,D**). Fura-2 ratio (F-ratio 340/380) was calculated and analyzed with NIS-Element AR 3.0 Software. *CPA*, is cyclopiazonic acid.

In order to further explore the mechanism of thrombin-induced EC permeability, we determined the role of BiP/GRP78 in mediating Ca^2+^ signaling, a critical determinant of EC permeability^14^. HPAEC were pretreated with SubAB, SubA or its inactive mutant SubA_A272_B and then loaded with Fura2-AM for 15 min. Ratiometric measurements of intracellular Ca^2+^ were made in response to thrombin during extracellular Ca^2+^ depletion-repletion conditions. Results indicate that while inactivation of BiP/GRP78 by SubAB completely blocked the store-operated Ca^2+^ entry from the extracellular medium (represented by 2^nd^ peak), it only partially inhibited Ca^2+^ release from the ER stores (indicated by the first peak) (Fig. 7A,B). In contrast, cells treated with SubA and SubA_A272_B showed no effect on both Ca^2+^ release and entry (Fig. 7A,B). In a related experiment, we also assessed the effect of SubAB on cyclopiazonic acid (CPA)-induced Ca^2+^ signaling. It should be noted that CPA, like thapsigargin, induces store depletion-mediated Ca^2+^ entry via inhibition of sarcoendoplasmic reticulum Ca^2+^ATPase (SERCA)^36^. We found that SubAB abolished store-operated Ca^2+^ entry but had no effect on Ca^2+^ release response caused by CPA (Fig. 7C,D). Together, these data indicate a novel role of BiP/GRP78 in the mechanism of store-operated Ca^2+^ entry.

## Discussion

In the present study, we have uncovered BiP/GRP78 as a critical determinant of lung vascular inflammation and injury via its ability to mediate EC permeability and inflammation. Using an aerosolized bacterial LPS inhalation mouse model of ALI, we found that inactivating BiP/GRP78 using SubAB protected against LPS-induced lung inflammation and injury and LPS-induced decrease in lung compliance. Introduction of BiP/GRP78-DN via cationic liposomes, which specifically target the lung endothelium for gene transfer^32^, also dampened LPS-induced proinflammatory gene expression and lung PMN infiltration, indicating a critical role of endothelial BiP/GRP78 in the mechanism of lung vascular inflammation. *In vitro* studies, using cultured EC revealed that thrombin induced an increase in BiP/GRP78 levels and pretreatment of cells with ER stress inhibitor 4-PBA prevented this response. Consistent with a role of BiP in EC inflammation^22^, 4-PBA also inhibited thrombin-induced activation of NF-κB and expression of proinflammatory genes, implicating a role of ER stress-BiP/GRP78 axis in EC inflammation. Our data also reveal a novel role of BiP/GRP78 in mediating EC permeability by virtue of activating Ca^2+^ signaling and promoting VE-cadherin disassembly. We further show that it is the intracellular BiP/GRP78 that mediates thrombin-induced EC inflammation and barrier disruption. Together, our data indicates that intracellular BiP/GRP78 contributes to ALI pathogenesis, at least in part, through its ability to induce inflammation and permeability in EC.

Alterations in pulmonary endothelium play a central role in the pathogenesis of several chronic and acute lung diseases. The main characteristics of pulmonary endothelial dysfunction are reflected by increased permeability leading to vascular leakage, edema formation, and acquisition of proinflammatory phenotype with increased expression of inflammatory mediators leading to PMN extravasation. In the present study, we analyzed the role of BiP/GRP78 in the pathogenesis of ALI in the context of pulmonary endothelium. Our results indicate that BiP/GRP78 contributes to lung vascular inflammation and injury. Intraperitoneal injections of BiP/GRP78 inhibitor SubAB decreased lung vascular injury caused by LPS. Moreover, pretreatment of mice with SubAB was associated with reduced lung PMN sequestration and migration caused by LPS. Consistent with this, LPS-induced levels of proinflammatory mediators such as adhesion molecules, cytokines, and chemokines, which are required for adhesion of PMN to the endothelium and subsequent extravasation of adherent PMN into the surrounding tissues, were reduced in the lungs of SubAB-treated mice compared to SubA_A272_B-treated mice. Importantly, LPS-induced decrease in lung compliance was protected in mice treated with SubAB but not in mice treated with SubA_A272_B. Our data highlight a novel role of BiP/GRP78 as a proinflammatory molecule in LPS-induced lung inflammation and injury.

Since BiP/GRP78 is an inherently prosurvival/antiapoptotic protein, it is expressed at high levels in the cytoplasm of cancer cells from where a small fraction of it translocates to the plasma membrane to serve as receptor, and is critical to tumor cell signaling and viability^23^. Recent studies have identified BiP/GRP78 on the surface of endothelial cells as well^37,38^. Hence, we analyzed the role of intracellular versus cell surface BiP/GRP78 in EC inflammation and barrier disruption using the holoenzyme SubAB and SubA. SubAB specifically cleaves and inactivates the global (intracellular and cell surface) pool of BiP/GRP78 whereas SubA cleaves only the cell surface BiP/GRP78^29^, as it lacks the B subunit required for internalization of the holoenzyme. Our data indicate that unlike cancer cells, only a small fraction of BiP/GRP78 is expressed on EC cell surface and it’s the intracellular BiP/GRP78 that mediates thrombin-induced EC inflammation and permeability. Furthermore, we demonstrate that BiP/GRP78 inactivation by SubAB protected against thrombin-induced EC permeability by initially decreasing the barrier disruption and later by increasing the barrier recovery. Mechanistically, we showed that BiP/GRP78 contributes to thrombin-induced EC permeability via VE-cadherin disassembly, a major mechanism of AJ disruptions and increased EC permeability^14,39,40^.

Activation of Ca^2+^ signaling is critical for EC permeability and inflammation. Studies have shown that thrombin binding to protease activated receptor-1 (PAR1) activates heterotrimeric G proteins, G_12_/G_13_ and G_q_ thereby mediating generation of inositol 1,4,5-trisphosphate (IP_3_), which in turn binds to inositol 1,4,5- trisphosphate receptor (IP_3_R) on ER, and signals Ca^2+^ release from ER stores. Depletion of ER Ca^2+^ induces activation of store-operated channels (SOC) at the plasma membrane, resulting in Ca^2+^ entry and refilling of ER stores^36^. Our data indicate that inactivation of BiP/GRP78 by SubAB abolished the store-depletion operated Ca^2+^ entry (SOCE) from the extracellular milieu but only partially inhibits the Ca^2+^ release in response to thrombin. SubAB also abolished the store-operated Ca^2+^ entry but had no effect on Ca^2+^ release response caused by CPA. Thus, these data identify a novel role of BiP/GRP78 in the mechanism of store-operated Ca^2+^ entry. Stromal interacting molecule 1 (STIM1), an ER Ca^2+^ sensor, is an important regulator of store-operated Ca^2+^ entry^41^. Upon sensing depletion of ER Ca^2+^, STIM1 organizes into a puncta and translocates to the close proximity of plasma membranes where it interacts with Ca^2+^-selective Orai1 channel to induce store-operated Ca^2+^ entry in EC^42–45^. Given that both BiP/GRP78 and STIM1 are ER proteins, it is likely that BiP/GRP78 associates with STIM1 in the ER and that this interaction is vital for STIM1 function, and thereby store-operated Ca^2+^ entry; however, this possibility remains to be addressed.

Together, our data show that both EC inflammation and permeability are regulated by the intracellular BiP/GRP78 and not by cell surface BiP/GRP78. It should, however, be noted that other studies have shown that BiP/GRP78 functions as a cell surface receptor mediating barrier protective effects of oxidized phospholipids (OxPLs)^37^. It is likely that stimulation of EC with barrier protective agents such as OxPAPC results in the activation of cell surface BiP/GRP78 which in turn activates the anti-inflammatory signaling cascade, whereas agents such as thrombin, which are potent inducers of EC permeability, engage the intracellular pool of BiP/GRP78 to induce proinflammatory phenotype in EC. In view of these findings highlighting a role of BiP/GRP78 in EC inflammation and permeability, we investigated the contribution of endothelial BiP/GRP78 in lung inflammatory responses. We found that expression of BiP/GRP78-DN in the lung endothelium was effective in reducing the expression of pro-inflammatory mediators and lung PMN infiltration in mice challenged with LPS. These data underscore the importance of endothelial BiP/GRP78 in lung inflammatory injury^22^. However, it should be stressed that these data do not exclude the involvement of BiP/GRP78 in alveolar epithelial cells and macrophages in this model of ALI. The effects of SubAB in reducing the levels of IL-1β and MCP-1 that are also produced by epithelial and inflammatory cells besides EC support such a possibility. Thus, analyzing the specific contribution of BiP/GRP78 in endothelial vs. epithelial cells or macrophages in the context of ALI will require additional studies using mice with cell-specific deletion of BiP/GRP78.

## Materials and Methods

### Reagents

Human alpha thrombin was obtained from Enzyme Research Laboratories (South Bend, IN). Lipopolysaccharide (LPS) from *E*. *coli*, diethylaminoethyl (DEAE)-dextran, and 4-phenylbutyrate (4-PBA) were purchased from Sigma-Aldrich Chemical (St. Louis, MO). Polyclonal antibodies to VCAM-1, ICAM-1, IκBα, RelA/p65, and β-actin were from Santa Cruz Biotechnology (CA). Antibodies to VE-cadherin were purchased from Abcam (Cambridge, MA) and BD Biosciences (San Jose, CA). BiP/GRP78 polyclonal antibody was obtained from Cell Signaling Technology (Beverly, MA). Expression vector encoding Wild type BiP/GRP78 and dominant negative BiP/GRP78 were from Addgene. SubAB, SubA and the non-active derivative SubA_A272_B were purified as previously described^28,29,46^. All other materials were from VWR Scientific Products Corporation (Gaithersburg, MD) and Fisher Scientific (Pittsburgh, PA).

### Murine model of ALI

Male 8- to 10-wk-old wild-type (WT) C57BL/6 mice (Jackson, Bar Harbor, ME) were exposed to an aerosol of saline alone or saline containing *Escherichia coli* LPS (0.5 mg/ml, 6 ml) for 30 min in a chamber, as described^47^. All animal care and treatment procedures were approved by the University of Rochester Committee on Animal Resources and performed in accordance with National Institutes of Health guidelines.

### Measurement of lung inflammation and injury

Mouse lung homogenates were prepared in radioimmune precipitation (RIPA) buffer supplemented with protease inhibitor cocktail (Sigma-Aldrich) as described^17,18,47^. The levels of ICAM-1, IL-1β, albumin, and MCP-1 in BAL fluids were determined using ELISA kits from R&D Systems (Minneapolis, MN) as described^17,18^. VCAM-1 levels in lung homogenates were determined by ELISA as described^47^. The recruitment of PMN in the lung was determined by monitoring the myeloperoxidase activity in the lung tissues as described^17,18^.

Wet-to-dry lung weight ratio was measured in mice that were not subjected to BAL^48^. Blood was removed from the lungs by gentle infusion of 10 ml of phosphate-buffered saline containing 5 mM EDTA through the right ventricle. The lungs were excised en bloc and blotted dry, and the right lungs were snap-frozen in liquid nitrogen. The left lungs were weighed before and after being dried at 60 °C for 24 h to calculate lung wet-to-dry weight ratio^49^.

### Physiologic assessment of pulmonary function in live, ventilated mice

Dynamic lung compliance and lung resistance were measured in live ventilated mice using a whole-body plethysmograph (BUXCO Electronics, Wilmington, NC) connected to a Harvard rodent ventilator (Harvard Apparatus, South Natick, MA), as previously described^50^. Dynamic lung compliance was normalized to the peak body weight of each animal. Respiratory rates were measured using whole-body unrestrained chambers (BUXCO Electronics). Data were collected and analyzed using the Biosystems XA software package (BUXCO Electronics).

### Cationic liposome mediated gene transfer

Wild type C57BL/6 L mice were injected i.v. with empty vector (EV)/liposome complex or dominant negative mutant BiP/GRP78 (pCMV-BiP/GRP78-DN)/liposome complex. After 72 hours mice were aerosolized with 6 ml of saline alone or saline containing *E*.*Coli* LPS (0.5 mg/ml) for 30 min. Sixteen hours later lung homogenates were analyzed for inflammation and injury^32,47^.

### Endothelial cells

Human pulmonary artery endothelial cells (HPAEC) were obtained from Lonza (Walkersville, MD) and cultured in 2% gelatin coated flasks using endothelial basal medium 2 (EBM2) with bullet kit additives (BioWhittaker, Walkersville, MD),as described^47,51^. Experiments were performed in cells below *passage 6*.

### Immunoblot analysis

After appropriate treatments HPAEC were lysed in radioimmune precipitation (RIPA) buffer. Total cell lysates were analyzed on SDS-PAGE followed by transfer on to nitrocellulose membranes. The membranes were subsequently incubated with primary antibody overnight at 4 °C followed by secondary antibody incubation at room temperature for 1 h. Subsequently the blot was developed using an enhanced chemiluminescence (ECL) method, as described^18^. Blots shown in the result section may have come from the membrane with more samples in various groups.

### Reporter gene constructs and luciferase assay

The construct pNF-κB-LUC containing five copies of consensus NF-κB sequences linked to a minimal E1B promoter-luciferase gene was purchased from Stratagene (La Jolla, CA). Reporter gene transfections and luciferase assays were performed essentially as described^47^. Briefly, 5 µg of DNA was mixed with DEAE-dextran (50 µg/ml) in serum-free EBM2, and the resulting mixture was applied onto cells that were 60–80% confluent. 0.125 µg of pTKRLUC plasmid (Promega, Madison, WI) containing *Renilla* luciferase gene driven by the constitutively active thymidine kinase promoter was used to normalize the transfection efficiencies. After 1 h, cells were exposed to 10% DMSO in serum-free EBM2 for 4 min and then washed twice with PBS and allowed to grow to confluency in EBM2–10% FBS. After appropriate treatments, cells were lysed in passive reporter lysis buffer (Promega) and cell extracts were assayed for firefly and *Renilla* luciferase activities using dual luciferase reporter assay system (Promega).The data were expressed as a ratio of firefly to *Renilla* luciferase activity.

### ELISA

Cytokines (IL-6 and IL-1β) and chemokines (IL-8 and MCP-1) levels in HPAEC culture supernatants were determined using ELISA kits from R&D Systems (Minneapolis, MN) according to the manufacturer’s recommendations^47^.

### Isolation of cell surface proteins

Cell surface proteins were isolated as per manufacturer’s instructions (ab 206998). Confluent EC were rinsed twice with PBS and then covered with ice-cold Sulfo-NHS-SS-Biotin solution. The plates were gently shaken at 4 °C for 30 minutes and the reaction was stopped using quenching solution. Cells were gently scraped and lysed with radioimmune precipitation buffer. To purify surface proteins, the lysate was mixed with Streptavidin beads and incubated at room temperature for 1.5 hours. The bound cell surface proteins were eluted in 100 ul of elution buffer containing DTT and the proteins in the eluate were then separated on a SDS-PAGE for further analysis.

### Immunofluorescence

HPAEC grown to confluence on 2% gelatin-coated coverslips were subjected to immunofluorescence staining as per the protocol described^51^. Polyclonal antibody from BD Biosciences was used to visualize VE-cadherin. Nuclei were visualized using DAPI. Following staining the coverslips were rinsed in PBS and mounted on the slide using Vectashield mounting media (Vector Laboratories, Lincolnshire, IL).Images were obtained using a Nikon fluorescence microscope (Nikon Instech, Tokyo, Japan).

### *In vitro* measurement of endothelial permeability by transendothelial electrical resistance (TER)

Electrical cell-substrate impedance sensing (ECIS) system from Applied Biophysics, Troy, NY was used to monitor TER, a measure of endothelial barrier integrity, across confluent EC monolayer as described^13^. Briefly, HPAEC were grown to confluence on 2% gelatin coated gold microelectrodes in EBM2 containing 10% FBS and bullet kit additives. 24 h later the complete culture medium was replace with EBM2 containing 1% FBS. After 2 h the cells were treatment as per the experimental requirement followed by thrombin treatment. The TER was monitored for 4–6 h and was normalized to the initial voltage and expressed as a fraction of the normalized resistance value^47^.

### Cytosolic Ca^2+^ measurements in intact cell population

HPAEC grown to confluence on 25 mm glass coverslips (Fisher Scientific) were loaded with 2 µM Fura-2 AM (Invitrogen), a cell permeable fluorescent probe used for measuring change in cytosolic Ca^2+^, for 30 minutes. After dye loading, the cells were washed twice with Ca^2+^ free HBSS buffer, and the coverslips were mounted on an inverted microscope (Eclipse TE2000-E, Nikon). Calcium release from the intracellular stores was determined by perfusing Ca^2+^ free imaging buffer and stimulating cells with thrombin (2.5U). Store-operated Ca^2+^ entry was measured, following addition of 1.26 mM Ca^2+^. Images were captured at 510 nm using a digital CCD camera (CoolSNAP HQ2, Photometrics) and an imaging software (NIS-Element AR 3.0, Nikon) after alternating excitations at 340 and 380 nm. Fura-2 ratio (F-ratio 340/380) was calculated and analyzed later offline with NIS-Element AR 3.0 Software.

### Cell adhesion assay

HPAEC grown to confluence in a 96 well black polystyrene plates (Corning), were subjected to adhesion assay as described^35^. HL-60 cells were labelled with 2.5 µM Calcein-AM (Invitrogen) for 30 minutes. 200 µl of Calcein-AM loaded HL-60 cells were added to each well and incubated for 1 hour at 37 °C. After 1 hour the non-adherent cells were removed by gentle wash with Phenol Red free DMEM media. Fluorescence was measured using a fluorescent plate reader at an excitation of 495 nm as described^35^.

### Statistical analysis

Standard one-way ANOVA was used to analyze the results, which were presented as mean ± SE. Tukey’s test (Prism 5.0, GraphPad Software, San Diego) was used to determine the significance between the groups. A *P* value < 0.05 between two groups was considered statistically significant^47^.
